# (*Z*)-*N*-(2,6-Diiso­propyl­phen­yl)-1-[(2-meth­oxyphen­yl)amino]­methanimine oxide

**DOI:** 10.1107/S241431462400988X

**Published:** 2024-10-21

**Authors:** David O. Juma, Bernard Omondi, Sizwe J. Zamisa, Wisdom Munzeiwa

**Affiliations:** a School of Chemistry and Physics, University of KwaZulu Natal, Private Bag X54001, Westville, Durban, 4000, South Africa; b Chemistry Department, Bindura University of Science Education, Private Bag 1020, Bindura, Zimbabwe; Universitat de lesIlles Balears, Palma de Mallorca, Spain

**Keywords:** crystal structure, zwitterion, non-symmetric hy­droxy­formamidine

## Abstract

The mol­ecular structure of the title compound, C_20_H_26_N_2_O_2_ reveals non-co-planarity between the central formamidine backbone and each of the outer meth­oxy- and *i*-propyl- substituted benzene rings with dihedral angles of 7.88 (15) and 81.17 (15)°, respectively, indicating significant twists in the mol­ecule. In the crystal, inter­molecular C—H⋯O inter­actions, forming an *R*^3^_4_(30) graph set, occur within a two-dimensional layer that extends along the *ac* plane.

## Abstract 2

The crystal structure of a zwitterionic, non-symmetric hy­droxy­formamidine derivative is described.

## Graphical abstract

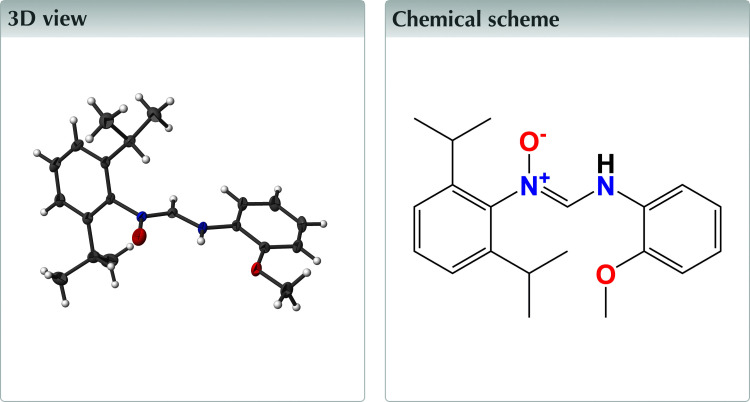

## Structure description

The tittle compound is a member of the formamidine class of compounds, which follow the general structure *R*N—C(*R*′)=N*R*′′, where *R*, *R*′ and *R*′′ can represent either hydrogen, alkyl or aryl groups (Zamisa *et al.*, 2021 ▸; Barker & Kilner, 1994 ▸). Their varied structures have led to investigations into their potential medicinal uses, uncovering properties such as anti­microbial and anti­cancer activities (Clement, 2002 ▸; Stojak *et al.*, 2014 ▸). Recently, we focused on the application of formamidine metal complexes as catalysts in ring-opening polymerization reactions (Munzeiwa *et al.*, 2018 ▸). As part of our work to develop new derivatives with superior catalytic abilities, we synthesized the title compound and analysed its crystal structure.

**Figure 1:**
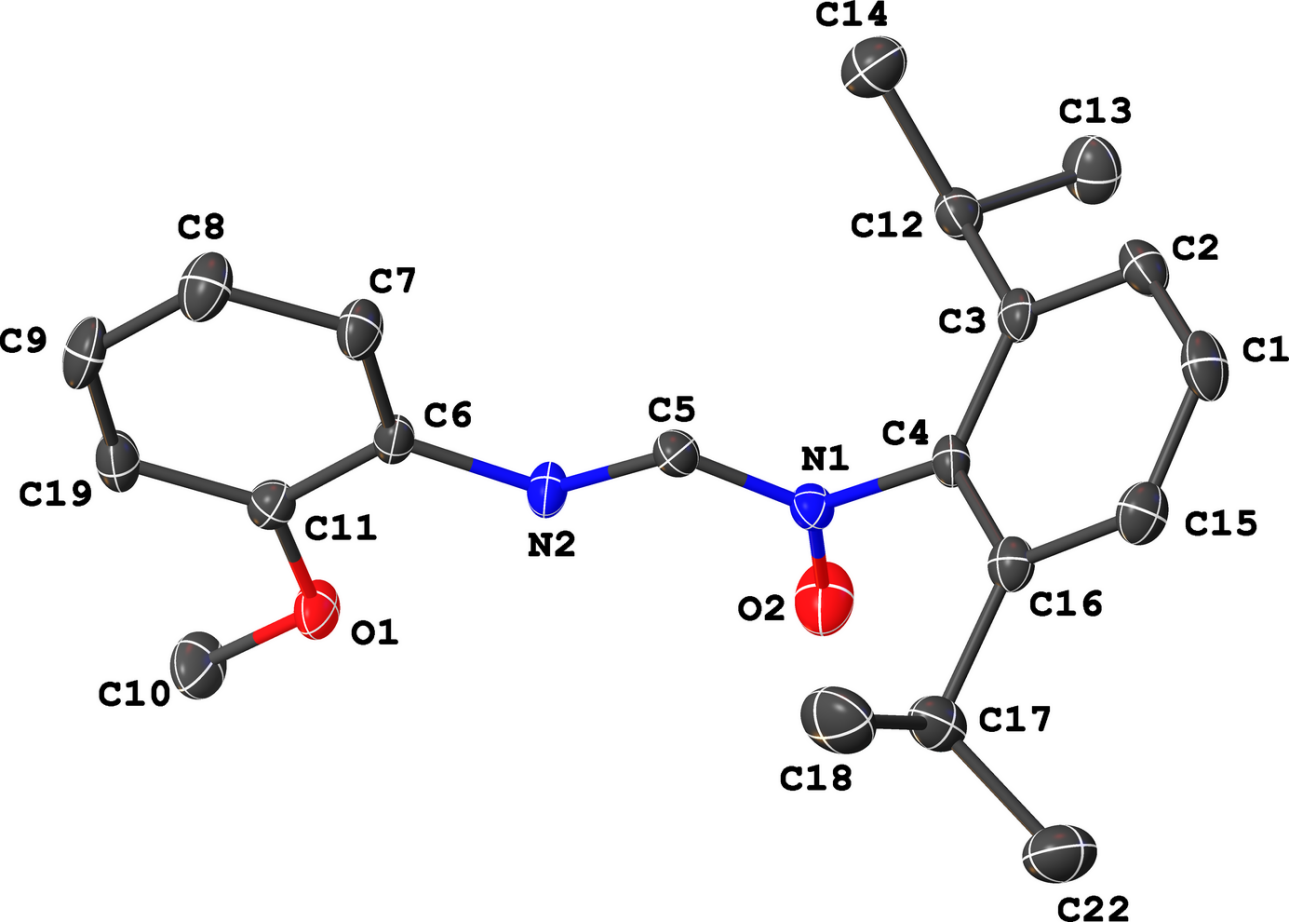
Mol­ecular structure of the title compound showing the atom-numbering scheme and displacement ellipsoids drawn at the 50% probability level. All hydrogen atoms have been omitted for clarity.

The title crystal has one mol­ecule in the asymmetric unit, as shown in Fig. 1 ▸. The mol­ecular conformation of the title compound is described by a dihedral angle between the 2-meth­oxy­benzene plane and the formamidine backbone of 7.88 (15)°, while that between the 2,6-diiso­propyl­benzene plane and the backbone measures 81.17 (15)°, suggesting a notable twist of this plane relative to the backbone. Furthermore, the dihedral angle between the two benzene planes is 78.17 (6)°. All other intra­molecular bond parameters are comparable with those of (*Z*)-1-[(4-meth­oxy­phen­yl)amino]-*N*-phenyl­methanimine oxide (CSD refcode: GIKFUB; Giumanini *et al.*, 1999 ▸).

**Figure 2:**
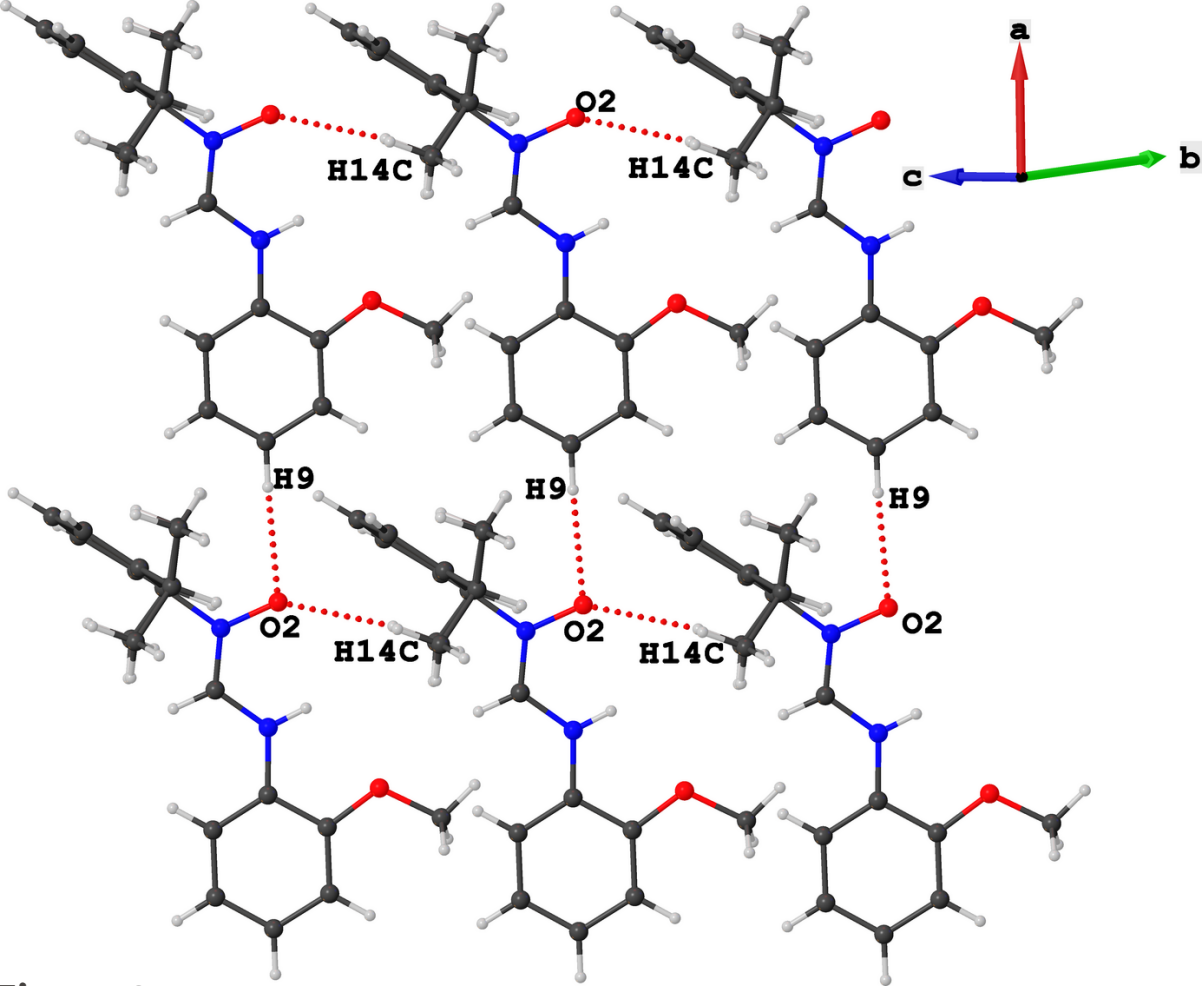
Representation of inter­molecular C—H⋯O inter­actions (red dotted bonds).

Inter­molecular C—H⋯O hydrogen bonds were found in the mol­ecular packing of the title compound, Table 1 ▸. The oxygen atom is involved in bifurcated C14—H14*C*⋯O2 and C9—H9⋯O2 inter­actions with the hydrogen (H14*C*) atom of the isopropyl substituent and the hydrogen (H9) atom of the 2-meth­oxy­phenyl ring, respectively. The former links neighbouring mol­ecules along [100] whilst the latter joins neighbouring mol­ecules along [001]. Collectively, the two types of C—H⋯O inter­actions can be described by an 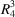
(30) graph set within a two-dimensional supra­molecular arrangement that propagates along the *ac* plane (Fig. 2 ▸).

## Synthesis and crystallization

The title compound was synthesized using a modified protocol (Munzeiwa *et al.*, 2017 ▸). Thus, *N*-(2,6-diiso­propyl­phen­yl)-*N*′-(2-meth­oxy­phen­yl)formamidine (1 mmol) was dissolved in di­chloro­methane (6 ml) followed by the addition of solid sodium hydrogen carbonate (1 mmol). The mixture was cooled to 0°C. A slight excess of *meta*-chloro­per­oxy­benzoic acid (1.2 mmol) in di­chloro­methane (6 ml) was then added dropwise, and the reaction mixture was allowed to gradually warm to room temperature while stirring for 1 h. The mixture was washed with 2 × 25 ml of 5% potassium carbonate solution. The combined organic layers were dried over anhydrous sodium sulfate, filtered and the solvent evaporated to yield a solid residue. The crude solid was subsequently recrystallized from its methanol solution to produce crystals suitable for X-ray diffraction.

## Refinement

Crystallographic data and structure refinement details are summarized in Table 2 ▸.

## Supplementary Material

Crystal structure: contains datablock(s) I. DOI: 10.1107/S241431462400988X/tk4110sup1.cif

Structure factors: contains datablock(s) I. DOI: 10.1107/S241431462400988X/tk4110Isup2.hkl

Supporting information file. DOI: 10.1107/S241431462400988X/tk4110Isup3.cml

CCDC reference: 2389590

Additional supporting information:  crystallographic information; 3D view; checkCIF report

## Figures and Tables

**Table 1:** Hydrogen-bond geometry (Å, °)

*D*—H⋯*A*	*D*—H	H⋯*A*	*D*⋯*A*	*D*—H⋯*A*
C9—H9⋯O2^i^	0.95	2.39	3.337 (2)	174
C14—H14*C*⋯O2^ii^	0.98	2.47	3.445 (2)	172

Symmetry codes: (i) 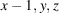; (ii) 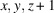.

**Table 2:** Experimental details

Crystal data	
Chemical formula	C_20_H_26_N_2_O_2_
*M*_r_	326.43
Crystal system, space group	Monoclinic, *P*2_1_/*c*
Temperature (K)	100
*a*, *b*, *c* (Å)	10.1243 (4), 23.941 (1), 7.5053 (3)
β (°)	91.315 (3)
*V* (Å^3^)	1818.70 (13)
*Z*	4
Radiation type	Mo *K*α
μ (mm^−1^)	0.08
Crystal size (mm)	0.22 × 0.18 × 0.11

Data collection	
Diffractometer	Bruker APEXII CCD
Absorption correction	Multi-scan (*SADABS*; Krause *et al.*, 2015 ▸)
*T*_min_, *T*_max_	0.984, 0.992
No. of measured, independent and observed [*I* > 2σ(*I*)] reflections	19835, 3268, 2304
*R*_int_	0.050
(sin θ/λ)_max_ (Å^−1^)	0.600

Refinement	
*R*[*F*^2^ > 2σ(*F*^2^)], *wR*(*F*^2^), *S*	0.044, 0.108, 1.03
No. of reflections	3268
No. of parameters	222
No. of restraints	1
H-atom treatment	H-atom parameters constrained
Δρ_max_, Δρ_min_ (e Å^−3^)	0.17, −0.34

Computer programs: *APEX2* and *SAINT* (Bruker, 2010 ▸), *SHELXT* (Sheldrick, 2015*a* ▸), *SHELXL2018/3* (Sheldrick, 2015*b* ▸) and *OLEX2* (Dolomanov *et al.*, 2009 ▸).

## full crystallographic data

### (Z)-N-(2,6-Diisopropylphenyl)-1-[(2-methoxyphenyl)amino]methanimine oxide Crystal data

**Table:**

C_20_H_26_N_2_O_2_	*F*(000) = 704
*M**_r_* = 326.43	*D*_x_ = 1.192 Mg m^−^^3^
Monoclinic, *P*2_1_/*c*	Mo *K*α radiation, λ = 0.71073 Å
*a* = 10.1243 (4) Å	Cell parameters from 3357 reflections
*b* = 23.941 (1) Å	θ = 2.6–26.1°
*c* = 7.5053 (3) Å	µ = 0.08 mm^−^^1^
β = 91.315 (3)°	*T* = 100 K
*V* = 1818.70 (13) Å^3^	Block, colourless
*Z* = 4	0.22 × 0.18 × 0.11 mm

### (Z)-N-(2,6-Diisopropylphenyl)-1-[(2-methoxyphenyl)amino]methanimine oxide Data collection

**Table:**

Bruker APEXII CCD diffractometer	2304 reflections with *I* > 2σ(*I*)
Graphite monochromator	*R*_int_ = 0.050
φ and ω scans	θ_max_ = 25.2°, θ_min_ = 1.7°
Absorption correction: multi-scan (SADABS; Krause *et al.*, 2015)	*h* = −12→12
*T*_min_ = 0.984, *T*_max_ = 0.992	*k* = −26→28
19835 measured reflections	*l* = −6→9
3268 independent reflections	

### (Z)-N-(2,6-Diisopropylphenyl)-1-[(2-methoxyphenyl)amino]methanimine oxide Refinement

**Table:**

Refinement on *F*^2^	1 restraint
Least-squares matrix: full	Hydrogen site location: inferred from neighbouring sites
*R*[*F*^2^ > 2σ(*F*^2^)] = 0.044	H-atom parameters constrained
*wR*(*F*^2^) = 0.108	*w* = 1/[σ^2^(*F*_o_^2^) + (0.0455*P*)^2^ + 0.5644*P*] where *P* = (*F*_o_^2^ + 2*F*_c_^2^)/3
*S* = 1.03	(Δ/σ)_max_ = 0.001
3268 reflections	Δρ_max_ = 0.17 e Å^−^^3^
222 parameters	Δρ_min_ = −0.34 e Å^−^^3^

### (Z)-N-(2,6-Diisopropylphenyl)-1-[(2-methoxyphenyl)amino]methanimine oxide Special details

**Table:**

Geometry. All esds (except the esd in the dihedral angle between two l.s. planes) are estimated using the full covariance matrix. The cell esds are taken into account individually in the estimation of esds in distances, angles and torsion angles; correlations between esds in cell parameters are only used when they are defined by crystal symmetry. An approximate (isotropic) treatment of cell esds is used for estimating esds involving l.s. planes.

### (Z)-N-(2,6-Diisopropylphenyl)-1-[(2-methoxyphenyl)amino]methanimine oxide Fractional atomic coordinates and isotropic or equivalent isotropic displacement parameters (Å^2^)

**Table:**

	*x*	*y*	*z*	*U*_iso_*/*U*_eq_	
O1	0.24618 (12)	0.68362 (5)	−0.12209 (16)	0.0240 (3)	
O2	0.63374 (13)	0.64315 (6)	0.11780 (18)	0.0331 (4)	
N1	0.58378 (14)	0.61829 (6)	0.25631 (19)	0.0170 (3)	
N2	0.37631 (14)	0.64203 (6)	0.1547 (2)	0.0190 (4)	
H2	0.413234	0.658969	0.064484	0.023*	
C1	0.82344 (18)	0.54648 (8)	0.6549 (3)	0.0260 (5)	
H1	0.874167	0.529441	0.748058	0.031*	
C2	0.77386 (18)	0.59966 (8)	0.6781 (2)	0.0225 (4)	
H2A	0.791133	0.618873	0.786910	0.027*	
C3	0.69884 (16)	0.62541 (7)	0.5438 (2)	0.0180 (4)	
C4	0.67049 (16)	0.59410 (7)	0.3911 (2)	0.0164 (4)	
C5	0.45720 (17)	0.61710 (7)	0.2758 (2)	0.0182 (4)	
H5	0.421147	0.598614	0.375576	0.022*	
C6	0.23759 (17)	0.64312 (7)	0.1612 (2)	0.0170 (4)	
C7	0.16819 (18)	0.62453 (8)	0.3053 (3)	0.0229 (4)	
H7	0.214562	0.610891	0.407812	0.028*	
C8	0.03063 (18)	0.62555 (8)	0.3021 (3)	0.0283 (5)	
H8	−0.016409	0.612074	0.401349	0.034*	
C9	−0.03726 (18)	0.64608 (8)	0.1551 (3)	0.0277 (5)	
H9	−0.131108	0.646543	0.152560	0.033*	
C10	0.1809 (2)	0.71154 (9)	−0.2667 (3)	0.0317 (5)	
H10A	0.120039	0.685618	−0.327481	0.048*	
H10B	0.246584	0.724767	−0.350876	0.048*	
H10C	0.131282	0.743447	−0.221269	0.048*	
C11	0.16860 (17)	0.66476 (7)	0.0128 (2)	0.0188 (4)	
C12	0.65352 (17)	0.68551 (8)	0.5626 (2)	0.0210 (4)	
H12	0.615423	0.697475	0.444555	0.025*	
C13	0.76943 (19)	0.72415 (8)	0.6074 (3)	0.0305 (5)	
H13A	0.805030	0.715203	0.726483	0.046*	
H13B	0.739196	0.763030	0.604830	0.046*	
H13C	0.838529	0.719026	0.519491	0.046*	
C14	0.5452 (2)	0.69169 (9)	0.6997 (3)	0.0320 (5)	
H14A	0.469131	0.668580	0.664185	0.048*	
H14B	0.517834	0.730900	0.706103	0.048*	
H14C	0.579024	0.679568	0.816829	0.048*	
C15	0.80004 (18)	0.51781 (8)	0.4976 (3)	0.0239 (5)	
H15	0.838574	0.482028	0.481773	0.029*	
C16	0.72077 (17)	0.54058 (7)	0.3619 (2)	0.0195 (4)	
C17	0.68975 (19)	0.50857 (8)	0.1912 (2)	0.0253 (5)	
H17	0.652702	0.535657	0.101812	0.030*	
C18	0.5848 (2)	0.46398 (9)	0.2228 (3)	0.0419 (6)	
H18A	0.620523	0.435607	0.304729	0.063*	
H18B	0.559595	0.446378	0.109107	0.063*	
H18C	0.506945	0.481362	0.274722	0.063*	
C19	0.03187 (17)	0.66612 (7)	0.0105 (3)	0.0233 (4)	
H19	−0.014889	0.680792	−0.090184	0.028*	
C22	0.8135 (2)	0.48265 (9)	0.1134 (3)	0.0371 (5)	
H22A	0.881168	0.511530	0.100393	0.056*	
H22B	0.791668	0.466460	−0.003513	0.056*	
H22C	0.847068	0.453307	0.193454	0.056*	

### (Z)-N-(2,6-Diisopropylphenyl)-1-[(2-methoxyphenyl)amino]methanimine oxide Atomic displacement parameters (Å^2^)

**Table:**

	*U*^11^	*U*^22^	*U*^33^	*U*^12^	*U*^13^	*U*^23^
O1	0.0185 (7)	0.0334 (8)	0.0201 (7)	0.0049 (6)	−0.0014 (6)	0.0075 (6)
O2	0.0261 (8)	0.0423 (9)	0.0311 (8)	−0.0019 (6)	0.0022 (6)	0.0131 (7)
N1	0.0163 (8)	0.0197 (8)	0.0150 (8)	0.0004 (6)	−0.0011 (6)	0.0019 (6)
N2	0.0129 (8)	0.0246 (8)	0.0194 (8)	0.0007 (6)	−0.0006 (6)	0.0055 (7)
C1	0.0223 (10)	0.0292 (11)	0.0260 (11)	−0.0024 (9)	−0.0074 (9)	0.0109 (9)
C2	0.0215 (10)	0.0279 (11)	0.0179 (10)	−0.0056 (8)	−0.0036 (8)	0.0027 (8)
C3	0.0113 (9)	0.0228 (10)	0.0199 (10)	−0.0026 (7)	0.0003 (8)	0.0036 (8)
C4	0.0120 (9)	0.0208 (10)	0.0164 (10)	−0.0012 (7)	−0.0017 (8)	0.0046 (8)
C5	0.0182 (10)	0.0208 (10)	0.0155 (10)	0.0015 (8)	−0.0013 (8)	0.0006 (8)
C6	0.0133 (9)	0.0162 (9)	0.0215 (10)	0.0008 (7)	−0.0010 (8)	−0.0018 (8)
C7	0.0182 (10)	0.0260 (11)	0.0245 (11)	0.0037 (8)	−0.0011 (8)	0.0067 (9)
C8	0.0197 (10)	0.0308 (11)	0.0346 (12)	−0.0001 (9)	0.0042 (9)	0.0088 (10)
C9	0.0125 (10)	0.0282 (11)	0.0423 (13)	0.0000 (8)	0.0008 (9)	0.0037 (10)
C10	0.0286 (11)	0.0422 (13)	0.0240 (11)	0.0090 (10)	−0.0028 (9)	0.0111 (10)
C11	0.0177 (10)	0.0186 (10)	0.0201 (10)	−0.0001 (8)	0.0013 (8)	−0.0005 (8)
C12	0.0204 (10)	0.0236 (10)	0.0189 (10)	0.0001 (8)	−0.0027 (8)	0.0004 (8)
C13	0.0273 (11)	0.0256 (11)	0.0385 (13)	−0.0039 (9)	−0.0030 (10)	−0.0012 (9)
C14	0.0298 (12)	0.0307 (12)	0.0357 (12)	0.0017 (9)	0.0059 (10)	−0.0029 (10)
C15	0.0220 (10)	0.0213 (10)	0.0283 (11)	0.0034 (8)	−0.0003 (9)	0.0062 (9)
C16	0.0163 (9)	0.0205 (10)	0.0218 (10)	−0.0019 (8)	0.0007 (8)	0.0035 (8)
C17	0.0310 (11)	0.0215 (10)	0.0232 (11)	0.0036 (8)	−0.0031 (9)	−0.0002 (8)
C18	0.0421 (14)	0.0421 (14)	0.0414 (14)	−0.0123 (11)	0.0004 (11)	−0.0132 (11)
C19	0.0189 (10)	0.0223 (10)	0.0284 (11)	0.0018 (8)	−0.0066 (9)	−0.0004 (9)
C22	0.0416 (13)	0.0368 (13)	0.0331 (12)	0.0053 (10)	0.0054 (10)	−0.0065 (10)

### (Z)-N-(2,6-Diisopropylphenyl)-1-[(2-methoxyphenyl)amino]methanimine oxide Geometric parameters (Å, º)

**Table:**

O1—C10	1.424 (2)	C10—H10B	0.9800
O1—C11	1.372 (2)	C10—H10C	0.9800
O2—N1	1.3095 (19)	C11—C19	1.384 (2)
N1—C4	1.445 (2)	C12—H12	1.0000
N1—C5	1.294 (2)	C12—C13	1.526 (3)
N2—H2	0.8800	C12—C14	1.529 (3)
N2—C5	1.349 (2)	C13—H13A	0.9800
N2—C6	1.407 (2)	C13—H13B	0.9800
C1—H1	0.9500	C13—H13C	0.9800
C1—C2	1.381 (3)	C14—H14A	0.9800
C1—C15	1.381 (3)	C14—H14B	0.9800
C2—H2A	0.9500	C14—H14C	0.9800
C2—C3	1.392 (2)	C15—H15	0.9500
C3—C4	1.394 (2)	C15—C16	1.394 (2)
C3—C12	1.518 (3)	C16—C17	1.519 (3)
C4—C16	1.398 (2)	C17—H17	1.0000
C5—H5	0.9500	C17—C18	1.529 (3)
C6—C7	1.377 (3)	C17—C22	1.526 (3)
C6—C11	1.401 (2)	C18—H18A	0.9800
C7—H7	0.9500	C18—H18B	0.9800
C7—C8	1.393 (3)	C18—H18C	0.9800
C8—H8	0.9500	C19—H19	0.9500
C8—C9	1.377 (3)	C22—H22A	0.9800
C9—H9	0.9500	C22—H22B	0.9800
C9—C19	1.390 (3)	C22—H22C	0.9800
C10—H10A	0.9800		
			
C11—O1—C10	116.96 (14)	C3—C12—C13	111.26 (15)
O2—N1—C4	119.89 (14)	C3—C12—C14	112.18 (15)
C5—N1—O2	120.10 (15)	C13—C12—H12	107.5
C5—N1—C4	119.96 (15)	C13—C12—C14	110.69 (16)
C5—N2—H2	117.4	C14—C12—H12	107.5
C5—N2—C6	125.18 (15)	C12—C13—H13A	109.5
C6—N2—H2	117.4	C12—C13—H13B	109.5
C2—C1—H1	119.7	C12—C13—H13C	109.5
C2—C1—C15	120.67 (17)	H13A—C13—H13B	109.5
C15—C1—H1	119.7	H13A—C13—H13C	109.5
C1—C2—H2A	119.6	H13B—C13—H13C	109.5
C1—C2—C3	120.72 (18)	C12—C14—H14A	109.5
C3—C2—H2A	119.6	C12—C14—H14B	109.5
C2—C3—C4	117.11 (16)	C12—C14—H14C	109.5
C2—C3—C12	120.91 (16)	H14A—C14—H14B	109.5
C4—C3—C12	121.96 (15)	H14A—C14—H14C	109.5
C3—C4—N1	118.05 (15)	H14B—C14—H14C	109.5
C3—C4—C16	123.60 (16)	C1—C15—H15	119.5
C16—C4—N1	118.35 (15)	C1—C15—C16	121.01 (17)
N1—C5—N2	120.13 (16)	C16—C15—H15	119.5
N1—C5—H5	119.9	C4—C16—C17	121.73 (16)
N2—C5—H5	119.9	C15—C16—C4	116.70 (17)
C7—C6—N2	123.31 (16)	C15—C16—C17	121.57 (16)
C7—C6—C11	119.35 (16)	C16—C17—H17	107.8
C11—C6—N2	117.34 (16)	C16—C17—C18	110.65 (16)
C6—C7—H7	119.7	C16—C17—C22	111.84 (16)
C6—C7—C8	120.58 (17)	C18—C17—H17	107.8
C8—C7—H7	119.7	C22—C17—H17	107.8
C7—C8—H8	120.0	C22—C17—C18	110.80 (17)
C9—C8—C7	120.06 (18)	C17—C18—H18A	109.5
C9—C8—H8	120.0	C17—C18—H18B	109.5
C8—C9—H9	120.1	C17—C18—H18C	109.5
C8—C9—C19	119.84 (17)	H18A—C18—H18B	109.5
C19—C9—H9	120.1	H18A—C18—H18C	109.5
O1—C10—H10A	109.5	H18B—C18—H18C	109.5
O1—C10—H10B	109.5	C9—C19—H19	119.9
O1—C10—H10C	109.5	C11—C19—C9	120.24 (17)
H10A—C10—H10B	109.5	C11—C19—H19	119.9
H10A—C10—H10C	109.5	C17—C22—H22A	109.5
H10B—C10—H10C	109.5	C17—C22—H22B	109.5
O1—C11—C6	115.16 (15)	C17—C22—H22C	109.5
O1—C11—C19	124.93 (16)	H22A—C22—H22B	109.5
C19—C11—C6	119.91 (17)	H22A—C22—H22C	109.5
C3—C12—H12	107.5	H22B—C22—H22C	109.5
			
O1—C11—C19—C9	−179.71 (17)	C4—C3—C12—C14	109.98 (19)
O2—N1—C4—C3	97.35 (19)	C4—C16—C17—C18	−102.6 (2)
O2—N1—C4—C16	−83.0 (2)	C4—C16—C17—C22	133.38 (18)
O2—N1—C5—N2	−0.8 (3)	C5—N1—C4—C3	−80.2 (2)
N1—C4—C16—C15	−177.70 (15)	C5—N1—C4—C16	99.5 (2)
N1—C4—C16—C17	1.8 (2)	C5—N2—C6—C7	8.4 (3)
N2—C6—C7—C8	−178.56 (17)	C5—N2—C6—C11	−172.09 (16)
N2—C6—C11—O1	−1.2 (2)	C6—N2—C5—N1	179.54 (16)
N2—C6—C11—C19	179.04 (16)	C6—C7—C8—C9	−1.0 (3)
C1—C2—C3—C4	3.5 (3)	C6—C11—C19—C9	0.0 (3)
C1—C2—C3—C12	−175.00 (17)	C7—C6—C11—O1	178.31 (16)
C1—C15—C16—C4	2.0 (3)	C7—C6—C11—C19	−1.4 (3)
C1—C15—C16—C17	−177.53 (17)	C7—C8—C9—C19	−0.4 (3)
C2—C1—C15—C16	−3.1 (3)	C8—C9—C19—C11	0.9 (3)
C2—C3—C4—N1	175.00 (15)	C10—O1—C11—C6	−173.25 (16)
C2—C3—C4—C16	−4.7 (3)	C10—O1—C11—C19	6.5 (3)
C2—C3—C12—C13	53.0 (2)	C11—C6—C7—C8	1.9 (3)
C2—C3—C12—C14	−71.6 (2)	C12—C3—C4—N1	−6.5 (2)
C3—C4—C16—C15	2.0 (3)	C12—C3—C4—C16	173.79 (16)
C3—C4—C16—C17	−178.50 (16)	C15—C1—C2—C3	0.2 (3)
C4—N1—C5—N2	176.73 (15)	C15—C16—C17—C18	76.9 (2)
C4—C3—C12—C13	−125.41 (18)	C15—C16—C17—C22	−47.1 (2)

### (Z)-N-(2,6-Diisopropylphenyl)-1-[(2-methoxyphenyl)amino]methanimine oxide Hydrogen-bond geometry (Å, º)

**Table:**

*D*—H···*A*	*D*—H	H···*A*	*D*···*A*	*D*—H···*A*
C9—H9···O2^i^	0.95	2.39	3.337 (2)	174
C14—H14*C*···O2^ii^	0.98	2.47	3.445 (2)	172

Symmetry codes: (i) *x*−1, *y*, *z*; (ii) *x*, *y*, *z*+1.
